# Identification of Bioactive Chemical Markers in *Zhi zhu xiang* Improving Anxiety in Rat by Fingerprint-Efficacy Study

**DOI:** 10.3390/molecules23092329

**Published:** 2018-09-12

**Authors:** Shao-Nan Wang, Yong-Sheng Ding, Xiao-Jie Ma, Cheng-Bowen Zhao, Ming-Xuan Lin, Jing Luo, Yi-Nan Jiang, Shuai He, Jian-You Guo, Jin-Li Shi

**Affiliations:** 1 School of Chinese Materia Medica, Beijing University of Chinese Medicine, 11A North Third Ring East Road, Chaoyang District, Beijing 100029, China; wangshaonanzz@163.com (S.-N.W.); 18811795287@163.com (Y.-S.D.); 20150931927@bucm.edu.cn (X.-J.M.); lovyeazhao@163.com (C.-B.Z.); linmingxuan@bucm.edu.cn (M.-X.L.); ljing314@163.com (J.L.); jiangyinan@bucm.edu.cn (Y.-N.J.); 20160931819@bucm.edu.cn (S.H.); 2 CAS Key Laboratory of Mental Health, Institute of Psychology, Chinese Academy of Sciences, 4A Datun Road, Chaoyang District, Beijing 100101, China

**Keywords:** anti-anxiety, *Valeriana jatamansi* Jones, fingerprint-efficacy relationship, PLSR

## Abstract

*Zhi zhu xiang* (ZZX for short) is the root and rhizome of *Valeriana jatamansi* Jones, which is a Traditional Chinese Medicine (TCM) used to treat various mood disorders for more than 2000 years, especially anxiety. The aim of the present work was to identify the bioactive chemical markers in *Zhi zhu xiang* improving anxiety in rats by a fingerprint-efficacy study. More specifically, the chemical fingerprint of ZZX samples collected from 10 different regions was determined by High Performance Liquid Chromatography (HPLC) and the similarity analyses were calculated based on 10 common characteristic peaks. The anti-anxiety effect of ZZX on empty bottle stimulated rats was examined through the Open Field Test (OFT) and the Elevated Plus Maze Test (EPM). Then we measured the concentration of CRF, ACTH, and CORT in rat’s plasma by the enzyme-linked immune sorbent assay (ELISA) kit, while the concentration of monoamine and metabolites (NE, DA, DOPAC, HVA, 5-HT, 5-HIAA) in the rat’s cerebral cortex and hippocampus was analysed by HPLC coupled with an Electrochemical Detector. At last, the fingerprint-efficacy study between chemical fingerprint and anti-anxiety effect of ZZX was accomplished by partial least squares regression (PLSR). As a result, we screened out four compounds (hesperidin, isochlorogenic acid A, isochlorogenic acid B and isochlorogenic acid C) as the bioactive chemical markers for the anti-anxiety effect of ZZX. The fingerprint-efficacy study we established might provide a feasible way and some elicitation for the identification of the bioactive chemical markers for TCM.

## 1. Introduction

Anxiety disorders are one of the most common mental disorders in modern society, which seriously affect people’s quality of life [1,2,3,4,5]. According to a recent study, the lifetime prevalence of anxiety disorders in Europe range from 19.3 to 28.8%, with an annual prevalence of 14% and an increasing trend [5]. However, researches on anxiety medications have been slow. At present, the commonly used drugs clinically are still benzodiazepines found in the mid-1950s [6]. As we all know, those drugs have obvious side effects, including withdrawal symptoms, sexual dysfunction, nausea and vomiting, lethargy, dizziness, sweating, dry mouth and elevated blood pressure [7,8,9]. It affects patients’ compliance and quality of life, so that patients cannot stick to treatment for a long time. As a result, more and more patients are looking for drugs and treatments with minor side effects.

Traditional Chinese Medicine (TCM) are gaining more and more attention, due to their lower side effects and long-standing clinical applications in treating anxiety disorders in China [10,11,12]. As a valuable TCM, *jatamansvaleriana* (*Zhi zhu xiang* in Chinese, ZZX for short), dried root and rhizome of *Valeriana jatamansi* Jones or *V. wallichii* DC, was first listed in Compendium of Materia Medica (BencaoGangmu). The 2015 edition of the Chinese pharmacopoeia records its effects of pain relief, diarrhea and sedation [13]. It is widely used to treat anxiety disorders in clinical prescription for more than 2000 years [14]. Recent studies have reported that ZZX and its iridoids have a significant anxiolytic effect [14,15,16,17,18,19].

Bioactive chemical markers is a new concept of quality control of TCM proposed by academician Liu Changxiao [20]. The basic conditions of bioactive chemical markers include: (1) The bioactive chemical markers are a chemical compound that formed in the process of preparation of Chinese medicinal materials and TCM products; (2) they are closely related to the efficacy properties of TCM and have clear chemical structures; and (3) qualitative and quantitative determination of bioactive chemical markers can be carried out. Selecting the appropriate bioactive chemical markers for monitoring the quality, efficacy, and safety is critical for efficient and reliable assessment of ZZX. Unfortunately, iridoids are not suitable for bioactive chemical markers of ZZX, because of its poor stability, and we can never achieve a real quality control in the absence of bioactive chemical markers.

In the previous study, our laboratory found that 35% ethanol extract of ZZX had a stable anti-anxiety effect [19]. This part of the extract contains a lot of phenolic acids, which exist widely in nature and have many pharmacological effects [21,22]. Hence, we selected 35% ethanol extract of ZZX to study the fingerprint-efficacy relationship, and to find the bioactive chemical markers of its anti-anxiety activity.

## 2. Results

### 2.1. HPLC

In order to achieve a stable and reproducible chemical fingerprint of ZZX, a comprehensive method validation on the developed HPLC-PDA fingerprint analysis was conducted. Results are shown in Table 1. According to the definite HPLC analysis conditions, the extract of the 10 batches of ZZX were examined. The chromatograms of ZZX were obtained.

The chromatographic fingerprints were automatically matched using Similarity Evaluation System for Chromatographic Fingerprint of Traditional Chinese Medicine (2012A Version, Committee of Chinese Pharmacopeia). The simulative mean chromatogram as a representative standard for 10 fingerprints was calculated and generated automatically by median method. Similarity values (>0.97) between each two chromatographic fingerprints were then determined using the above mentioned officially recommended software.

**Figure 1:**
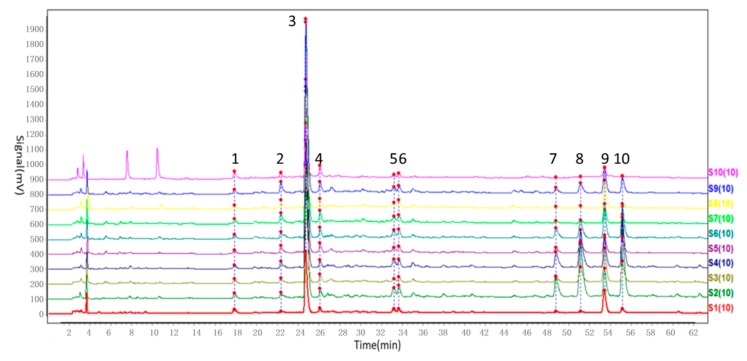
Fingerprint of ZZX. **1**: Neochlorogenic acid, **2**: Unknown compound X, **3**: Chlorogenic acid, **4**: Cryptochlorogenin acid, **5**: Unknown compound Y, **6**: Unknown compound Z, **7**: Isochlorogenic acid B, **8**: Isochlorogenic acid A, **9**: Hesperidin, **10**: Isochlorogenic acid C.

**Figure 2:**
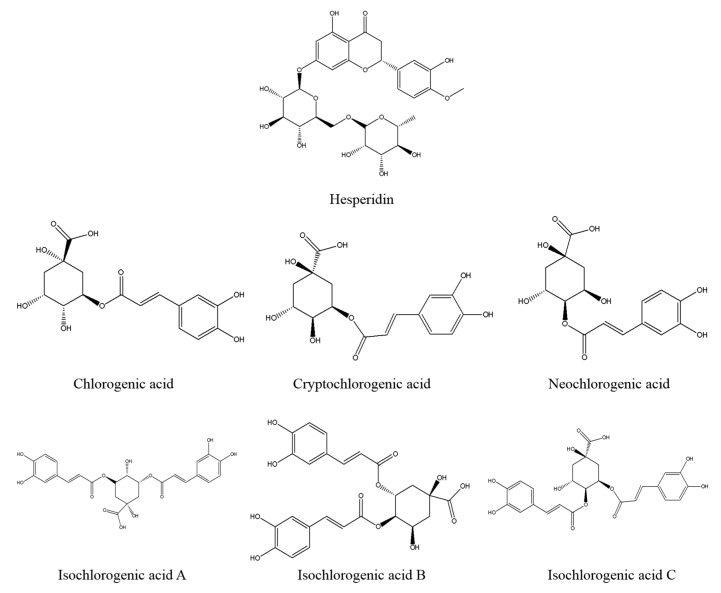
The chemical structural formulas of seven known compounds in ZZX.

We observed a total of 10 common peaks of the ZZX fingerprints with stable presence and higher response values (Figure 1 and Figure 2). By comparing their chromatograms with reference substances, seven of them were identified as neochlorogenic acid (**1**), chlorogenic acid (**3**), cryptochlorogenin acid (**4**), isochlorogenic acid B (**7**), isochlorogenic acid A (**8**), hesperidin (**9**), isochlorogenic acid C (**10**). Additionally, the contents of the seven identified compounds were determined by regression equation (Table 2), the other three compounds (number **2**, **5** and **6**), whose chemical structures have not been identified, were analyzed semi-quantitatively as peak areas (Table 3). In this study, we strictly controlled the injection volume and other conditions, so that the peak area could also represent the proportion of these compounds in ZZX of different regions.

### 2.2. Behavioral Tests

#### 2.2.1. Open-Field Test

**Figure 3:**
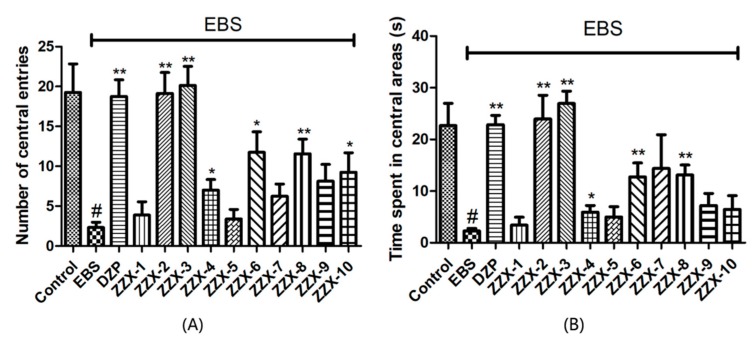
Effect of ZZX on the number of central entries and time spent in central areas in the open-field test in EBS rats. (**A**) Number of central entries; (**B**) time spent in central areas (s). Bars represent mean ± SEM. ^#^ represent *p* < 0.05 compared to control group; * represent *p* < 0.05 compared to EBS/ZZX group; ** represent *p* < 0.01 compared to EBS/ZZX group. One-way ANOVA with Student-Newman-Keuls post hoc test.

The one-way ANOVA revealed significant differences between groups in the number of central entries (F (12,96) = 9.213, *p* < 0.01, Figure 3), and time spent in central areas (F (12,96) = 11.331, *p* < 0.01, Figure 3). As shown in Figure 3, diazepam (1.0 mg/kg) significantly increased the number of central entries (*p* < 0.01) and the time spent in central areas (*p* < 0.01) compared with the EBS group. 

#### 2.2.2. Elevated Plus Maze

**Figure 4:**
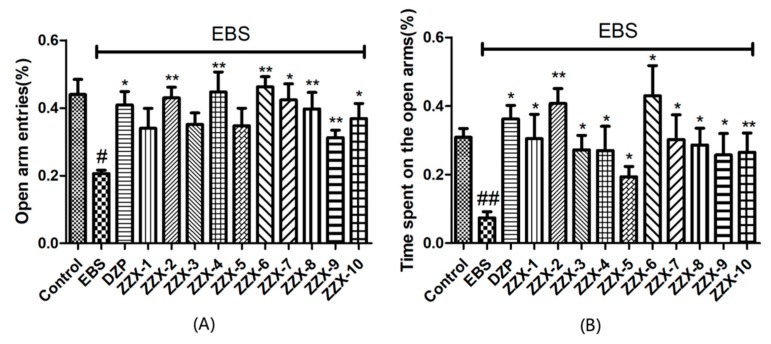
Effect of ZZX on the time spent on the open arms and the percentage of open arm entries in the elevated plus maze test in EBS rats. (**A**) Open arm entries (%); (**B**) time spent on the open arms (%). Bars represent mean ± SEM. ^#^ represent *p* < 0.05 compared to the control group; ^##^ represent *p* < 0.01 compared to the control group; * represent *p* < 0.05 compared to the EBS/ZZX group; ** represent *p* < 0.01 compared to the EBS/ZZX group. One-way ANOVA with Student-Newman-Keuls post hoc test.

The one-way ANOVA revealed significant differences between groups in the percentage of open arm entries (F (12,96) = 3.025, *p* < 0.01, Figure 4) and the time spent on the open arms (F (12,96) =4.013, *p* < 0.01, Figure 4). As shown in Figure 4, the EBS group significantly decreased the percentage of open arm entries (*p* < 0.05) and the time spent on the open arms (*p* < 0.01) of rats compared with the control group. The diazepam group had the opposite effect.

### 2.3. Serum CRF, ACTH, and CORT Levels

**Figure 5:**
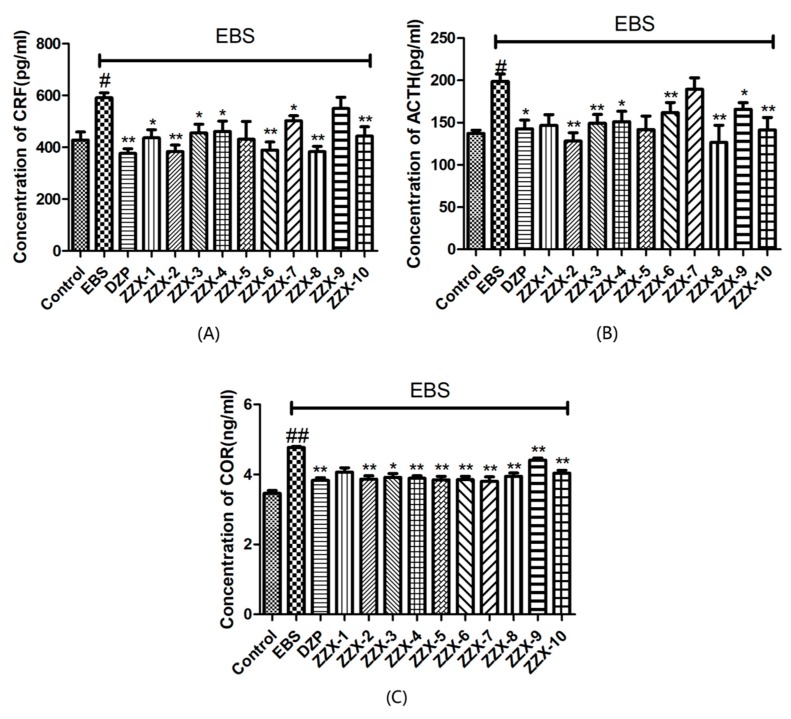
Effect of ZZX on serum CRF, ACTH and CORT levels after the behavior tests. (**A**) Concentration of CRF (pg/mL); (**B**) concentration of ACTH (pg/mL); (**C**) concentration of COR (ng/mL). Bars represent mean ± SEM. ^#^ represent *p* < 0.05 compared to the control group; ^##^ represent *p* < 0.01 compared to the control group; * represent *p* < 0.05 compared to the EBS/ZZX group; ** represent *p* < 0.01 compared to the EBS/ZZX group. One-way ANOVA with Student-Newman-Keuls post hoc test.

As shown in Figure 5, the one-way ANOVA indicated that there were significant differences in the contents of CRF (F (12,96) = 3.651, *p* < 0.01, Figure 5), ACTH (F (12,96) = 8.238, *p* < 0.01, Figure 5), and CORT (F (12,96) = 4.450, *p* < 0.01, Figure 5). After the empty bottle stimulation, the EBS groups significantly increased the level of CRF, ACTH and COR in rats’ plasma compared with the control groups (all *p* < 0.05). Compared with the EBS group, diazepam could decrease the level of CRF, ACTH, and COR in rats’ plasma (all *p* < 0.05). The levels of CRF, ACTH and COR in ZZX groups were different from each other. Among them, ZZX-2, 8 and 10 significantly reduced CRF, ACTH and COR levels in rat serum (*p* < 0.01), which was consistent with the behavioral results. 

### 2.4. Monoamine Neurotransmitters and Their Metabolites

In Table 4, the one-way ANOVA indicated that there were significant differences in the concentrations of monoamine neurotransmitters and their metabolites in the hippocampus of rats (F (12,96) = 3.540, *p* < 0.01, NE; F(12,96) = 3.801, *p* < 0.01, DOPAC; F (12,96) = 4.956, *p* < 0.01, DA; F(12,96) = 2.524, *p* < 0.05, 5-HIAA; F (12,96) = 3.571, *p* < 0.01, HVA; F (12,96) = 5.306, *p* < 0.01, 5-HT). Seen from the data, empty bottle stimulated rats could significantly increase the concentration of NE (*p* < 0.01), DOPAC (*p* < 0.05), DA (*p* < 0.01), 5-HIAA (*p* < 0.05), HVA (*p* < 0.01) and 5-HT (*p* < 0.01) in the hippocampus of the brain. DZP and ZZX-2, 3, 6, 8 could reverse it to a different extent.

In Table 5, the one-way ANOVA indicated that there were significant differences in the concentrations of monoamine neurotransmitters and their metabolites in the cerebral cortex of rats (F (12,96) = 2.980, *p* < 0.05, NE; F (12,96) = 2.962, *p* < 0.05, DA; F (12,96) = 1.461, *p* < 0.05, 5-HIAA; F (12,96) = 6.272, *p* < 0.01, 5-HT). The levels of NE (*p* < 0.05), DA (*p* < 0.05), 5-HT (*p* < 0.05) and 5-HIAA (*p* < 0.05) in EBS groups were significantly increased compared with the control groups. DZP and ZZX-2, 4, 6, 8 could decrease the content of NE, DA, DOPAC, HVA, 5-HT, and 5-HIAA in the rat’s cerebral cortex. There were no differences in the contents of DOPAC (F (12,96) = 1.885, *p >* 0.05) and HVA (F (12,96) = 1.594, *p >* 0.05). This part of data was not statistically analyzed in later experiments.

### 2.5. Discovery of the Bioactive Chemical Markers by PLSR 

**Figure 6:**
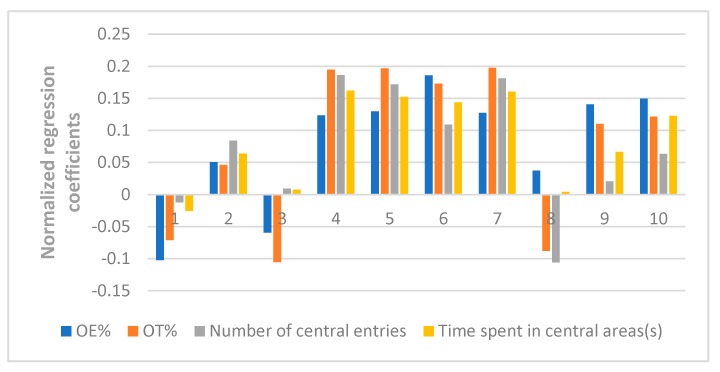
Normalized regression coefficients between the contents of the 10 ZZX samples and the values of the pharmacodynamic behavioral indices. **1**: Neochlorogenic acid, **2**: Chlorogenic acid, **3**: Cryptochlorogenin acid, **4**: Isochlorogenic acid B, **5**: Isochlorogenic acid A, **6**: Hesperidin, **7**: Isochlorogenic acid C, **8**: Unknown compound X, **9**: Unknown compound Y, **10**: Unknown compound Z. The regression coefficients were combined with the indices of the antianxiety effect, of which the regression coefficients of OE%, OT%, number of central entries and time spent in central areas were either positive, indicating a positive effect on antianxiety effect; or negative, indicating a negative effect on the antianxiety effect.

**Figure 7:**
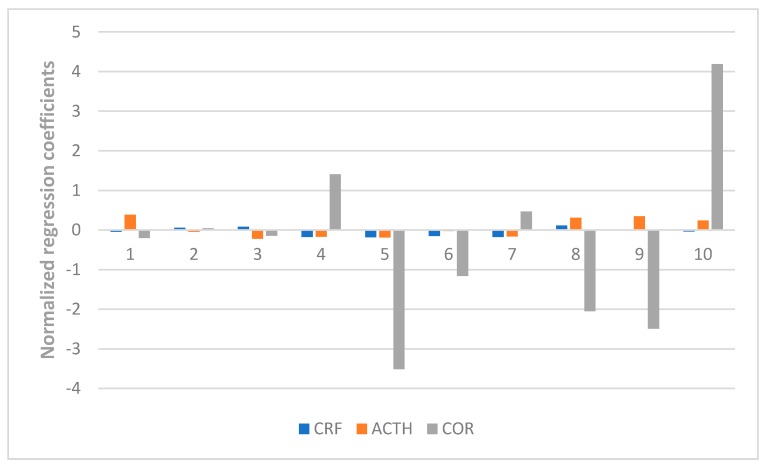
Normalized regression coefficients between the contents of the 10 ZZX samples and the concentration of CRF, ACTH, CORT in serum. **1**: Neochlorogenic acid, **2**: Chlorogenic acid, **3**: Cryptochlorogenin acid, **4**: Isochlorogenic acid B, **5**: Isochlorogenic acid A, **6**: Hesperidin, **7**: Isochlorogenic acid C, **8**: Unknown compound X, **9**: Unknown compound Y, **10**: Unknown compound Z. The regression coefficients were combined with the indices of the anti-anxiety effect, of which the regression coefficients of CRF, ACTH, COR were either positive, indicating a negative effect on anti-anxiety effect; or negative, indicating a positive effect on the anti-anxiety effect.

**Figure 8:**
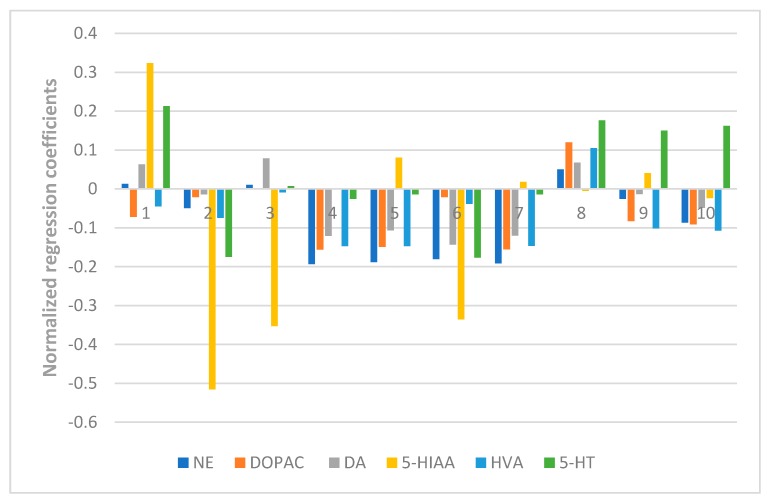
Normalized regression coefficients between the contents of the 10 ZZX samples and the content of monoamine neurotransmitters and their metabolites in the hippocampus of rats. **1**: Neochlorogenic acid, **2**: Chlorogenic acid, **3**: Cryptochlorogenin acid, **4**: Isochlorogenic acid B, **5**: Isochlorogenic acid A, **6**: Hesperidin, **7**: Isochlorogenic acid C, **8**: Unknown compound X, **9**: Unknown compound Y, **10**: Unknown compound Z. The regression coefficients were combined with the indices of the anti-anxiety effect, of which the regression coefficients of NE, DOPAC, DA, 5-HIAA, HVA, 5-HT were either positive, indicating a negative effect on anti-anxiety effect; or negative, indicating a positive effect on the anti-anxiety effect.

**Figure 9:**
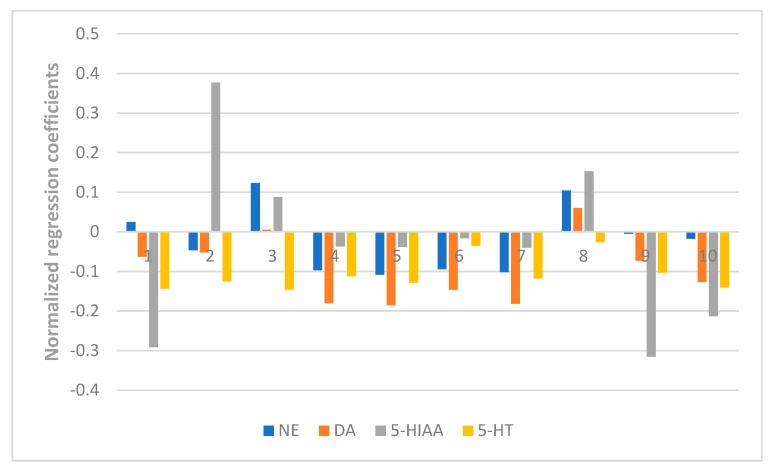
Normalized regression coefficients between the contents of the 10 ZZX samples and the content of monoamine neurotransmitters and their metabolites in the whole cerebral cortex of rats. **1**: Neochlorogenic acid, **2**: Chlorogenic acid, **3**: Cryptochlorogenin acid, **4**: Isochlorogenic acid B, **5**: Isochlorogenic acid A, **6**: Hesperidin, **7**: Isochlorogenic acid C, **8**: Unknown compound X, **9**: Unknown compound Y, **10**: Unknown compound Z. The regression coefficients were combined with the indices of the anti-anxiety effect, of which the regression coefficients of NE, DA, 5-HIAA, 5-HT were either positive, indicating a negative effect on anti-anxiety effect; or negative, indicating a positive effect on anti-anxiety effect.

Here in our research, a PLSR model was used to correlate the chromatographic data of ZZX and their pharmacodynamics indices, including the behavioral anti-anxiety indicators, contents of CRF, ACTH, CORT in serum, and the contents of monoamines and metabolites (NE, DA, DOPAC, HVA, 5-HT, 5-HIAA) in the brain. The X matrix of dimensions (10 × 10) from seven compounds and three common peak areas and their respective Y matrices of the different pharmacodynamics indices were used. The PLSR model evaluation was carried out from the perspective of total probability and multiple correlation coefficient. In this experiment, the probability values of each PLSR model were all less than 0.05 and the multiple correlation coefficient was greater than 0.9. Further, the normalized correlation coefficients of each compound to each pharmacodynamic indicator were listed in Figure 6, Figure 7, Figure 8 and Figure 9.

It can be seen clearly from Figure 6 that the normalized correlation coefficients of hesperidin, isochlorogenic acid A, isochlorogenic acid B, isochlorogenic acid C and unknown compound Z were much larger than other compounds. In the PLSR model, the greater the absolute value of regression coefficient, the greater the influence of this compound on the pharmacodynamic indicator. Moreover, a positive number represented that the content of the compound had positive correlation with the pharmacodynamic indicator, and a negative number represented the opposite effect. Thus, we preliminarily concluded that the hesperidin, isochlorogenic acid A, isochlorogenic acid B, isochlorogenic acid C and unkown compound Z were more responsible for the measured anti-anxiety activities.

As shown in Figure 7, isochlorogenic acid A and hesperidin had the most positive effects on the reduction of the concentration of CRF, ACTH, CORT in serum. Figure 8 and Figure 9 showed that hesperidin, isochlorogenic acid A, isochlorogenic acid B, isochlorogenic acid C had the most negative effects on the reduction of the concentration of monoamines and metabolites in brain. To sum up, the anti-anxiety effect of ZZX is achieved through the synergistic effect of multiple compounds on multiple pathways. Among them, hesperidin and isochlorogenic acid A, isochlorogenic acid B, isochlorogenic acid C are selected as the bioactive chemical markers, because of their obvious positive effect on the behavioral antianxiety indices. However, chlorogenic acid and unknown compound Z also hasdsome positive effects on the above indices, and follow-up studies are needed to demonstrate its anti-anxiety effect and mechanism.

## 3. Discussion

In our study, the chemical fingerprint of ZZX samples collected from 10 different regions was determined by HPLC and the similarity analyses were calculated based on 10 common characteristic peaks. In view of the similarity values among ZZX samples, all of them were higher than 0.97, which seemed to indicate that there were very little chemical differences among ZZX samples.

The results also showed the anti-anxiety effect of ZZX varies according to different groups. ZZX-2, 3 and 8 significantly increased the number of central entries and the time spent in central areas compared with the EBS group. ZZX-2 significantly increased the percentage of open arm entries and the time spent on the open arms of rats compared with the EBS group. Then we measured the concentration of CRF, ACTH, and CORT in rat’s plasma by the ELISA kit, while the concentration of monoamine and metabolites (NE, DA, DOPAC, HVA, 5-HT, 5-HIAA) in rat’s cerebral cortex and hippocampus was analysed by HPLC coupled with an Electrochemical Detector. ZZX-2, 8 and 10 significantly reduced CRF, ACTH and COR levels in rat serum. ZZX-2, 6, 8 could significantly decrease the concentration of NE, DA, 5-HIAA and 5-HT both in the hippocampus and cerebral cortex of the brain.

The inconsistency between the chemical information and bioactive information demonstrated that the similarity values of chromatographic fingerprints alone did not represent the anti-anxiety effect of ZZX. This is another proof of the need for our study. 

At last, the fingerprint-efficacy study between the chemical fingerprint of ZZX and its anti-anxiety effect was accomplished by PLSR. The normalized correlation coefficients of each compound to each pharmacodynamic indicator were obtained. We selected the top four compounds that had a significant impact on anti-anxiety indexes as bioactive chemical markers of ZZX. They are hesperidin, isochlorogenic acid A, isochlorogenic acid B and isochlorogenic acid C. Their content ranges from 10 different batches of ZZX are hesperidin (0.0639–0.3022%), isochlorogenic acid A (0.0070–0.5982%), isochlorogenic acid B (0.0153–0.1905%) and isochlorogenic acid C (0.00001–0.5713%). We surmise that this is the cause of the inconsistency in its anti-anxiety effect.

It is found that these compounds have anti-anxiety effects through literature studies. Hesperidin belongs to the dihydrobrass glycosides, and its structure can be regarded as the hydrogenation of the 2,3-digit double bonds of the basic nucleus of flavone. In recent years, the anti-anxiety effect of natural flavonoids has attracted many scholars’ attention [23,24,25,26]. Its anti-anxiety effect may be related to their common structural characteristics: with hydroxyl in the 5,7 positions [27]. In addition to hesperidin, other bioactive chemical markers are all phenolic acids. Phenolic acids exist widely in nature and have many pharmacological effects [21,22]. Stalmach A. and other researchers have shown that chlorogenic acid is absorbed into the blood in only about 30% of the small intestine, while 70% are absorbed by intestinal flora after metabolism [28]. Its main intestinal metabolite, caffeic acid, has anti-depressive and anti-anxiety effects [29,30,31]. Therefore, we speculate that the anti-anxiety effect of chlorogenic acid in ZZX may be related to their intestinal metabolites.

At present, the typical method of finding bioactive chemical markers in TCM usually requires the systematic separation of compounds. This methodology generally has the following shortfalls, such as being time and labor consuming. Even if the compounds in TCM are successfully separated, too many chemical compounds will still result in a huge amount of bioactivity screening, which is difficult to complete [32]. To solve these problems, some scholars have proposed the screening of bioactive chemical markers by spectral efficiency association method [20,33], and then choosing the appropriate stechiometry technology becomes the key to the feasibility of this theory. The current analysis methods of spectral efficiency association analysis mainly include: Grey relational analysis, Multi-linear regression analysis, PLSR and so on [34].

PLSR is developed on the basis of MLR and PCR. It is a classic modeling method and is almost a reference standard method, which is used in many commercial softwares (such as SIMCA, TQ analyst and Minitab) [34]. This method can extract the partial least squares component from the data of independent variables and the dependent variable data. This can effectively reduce the variable dimension of the original data. At the same time, the multiple correlation among the independent variables has been eliminated, making the modeling results dramatically improve the reliability and accuracy. PLSR is an ideal modeling method for processing high-dimensional small samples. In addition, the model based on PLSR is relatively simple. It contains all the original independent variables, and the regression coefficients of corresponding independent variables are easy to explain. It is widely used in the research of TCM spectrum-effect correlation analysis [35]. Hence PLSR was applied to rapidly screen bioactive chemical markers in our research.

Additionally, the selection of representative pharmacodynamic indicators is another determinant of the spectral efficiency association method. ZZX has been widely used to treat anxiety disorders in clinical prescription for more than 2000 years [14]. Recent studies have also reported that ZZX and its compounds have a significant anxiolytic effect [14,15,16,17,18,19]. Therefore, our research selects the anti-anxiety effect of ZZX as the pharmacodynamic indices.

Rodents are naturally interested in exploring the unknown new environment. Simultaneously, they tend to avoid potential threat therein. This conflict can cause contradictory psychological stress in rats and can simulate anxiety-like symptoms. For preclinical anxiety research, there are two mature and classic methods based on this characteristic of rodents—open-field test (OFT) and elevated plus-maze test (EPM) [36]. These two simple tests have been used in more than half of the rodent-based experiments since they were invented in the 1980s [37] and continue to be popular. So these two tests were selected to evaluate the anti-anxiety effect of ZZX in our research. And diazepam was selected as a positive control drug due to its determined curative effect [38].

As an important part of the neuroendocrine system, the hypothalamus-pituitary-adrenal axis (HPA) system is composed of the hypothalamus, pituitary and adrenal gland [39,40]. The activation of HPA axis is an important mechanism by which the brain reacts to acute or chronic stress such as anxiety [41,42]. In addition, it has been reported that ZZX extract plays a role in anti-anxiety via regulation of the HPA axis [43].

Research has shown that [44] stress can activate corticotropin releasing factor (CRF), the first hormone of HPA axis, and that CRF could induce the production of Pro-opiomelanocortin (POMC) via its receptor-mediated camp pathway. Then Adrenocorticotropic Hormone (ACTH) is released from the hypophysis. ACTH can promote the synthesis and release of corticosteroids in the adrenal cortex in the short term, while the long term effect involves the release of corticosteroids. Finally, corticosterone can change the structure of the prefrontal cortex and hypothalamus, affect cognitive, learning and memory function, and selectively damage the hippocampus, resulting in the hyperfunction of the hpa axis [45,46,47,48,49]. In addition, there is a peak in corticosterone secretion which occurs 5 to 10 min after exposure to an anxiety test [50]. At the same time, the central monoaminergic system (including NE, DA and 5-HT) has also been closely related to the pathophysiology and treatment of mental disorders [51,52]. Abnormal elevation of monoamine neurotransmitters in the cerebral cortex and hippocampus can induce neuropsychiatric disorders, especially anxiety [53,54]. Therefore, the change of hormone content (CRF, ACTH, CORT) in the HPA axis and the central monoaminergic system (including NE, DA and 5-HT) can be used as an important index for rating anxiety disorder and anxiety-like behavior.

This is the first time that hesperidin, isochlorogenic acid A, isochlorogenic acid B and isochlorogenic acid C were found to have a close relationship with the measured anti-anxiety activities in ZZX. Although such compounds have been found to have anti-anxiety effects through literature studies, there are few reports on this aspect. In addition, it’s not clear whether the prototyping of these compounds works or whether their metabolites do. Further studies are still needed to determine the anti-anxiety effect of them. But we can still come to the conclusion that the content of four target compounds is closely related to the anti-anxiety effect of ZZX. This makes them qualify for the bioactive chemical markers of ZZX. Considering its anti-anxiety effect, we suggest that the quality standard of ZZX can increase the content control of these four compounds: hesperidin (≥0.16%), isochlorogenic acid A (≥0.08%), isochlorogenic acid B (≥0.04%) andisochlorogenic acid C (≥0.08%).

However, there are still some limitations in our study. For example, the selected compounds should also be tested in order to find a possible correlation. The next step of our study is to blend the selected compounds by Uniform Design Method and observe the different anti-anxiety effects on various pharmacodynamics indices. The PLSR model will be used to find a possible correlation and we are now undertaking this work. Although our study has some limitations, it is still a promising method to find bioactive chemical markers in TCM by means of fingerprint-efficacy method.

## 4. Materials and Methods

### 4.1. The Complete Procedure of Our Strategy

**Figure 10:**
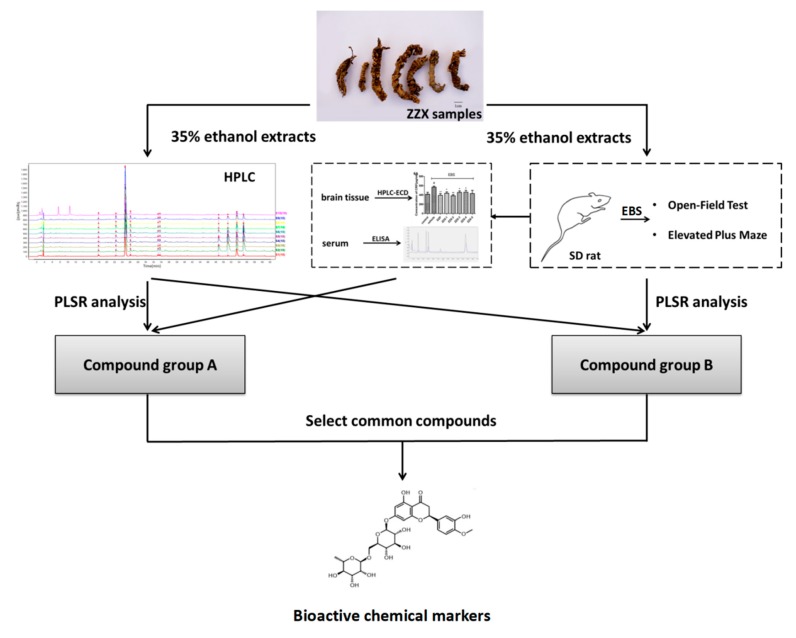
The overall flow chart of the experiment.

The complete procedure of our strategy is shown in Figure 10. Firstly, the chemical fingerprints of ZZX samples were determined by high performance liquid chromatography coupled with photodiode array detector (HPLC-PDA), then the similarity analyses were calculated and common characteristic peaks were identified. Secondly, anti-anxiety effects of ZZX samples were tested by open field test (OFT) and elevated plus maze (EPM) on empty bottle stimulated rats, which is widely used in anti-anxiety researches [55,56]. Thirdly, the relationship between the anti-anxiety effects of ZZX samples and the common compounds in it were studied by partial least squares regression (PLSR), and the bioactive chemical markers were screened out. Our strategy provided a feasible way and some elicitation for the quality control of ZZX and with the other TCM.

### 4.2. Materials and Reagents

The samples of ZZX were collected from 10 different regions of China (Table 6). The voucher specimens were identified by Prof. Jin-Li Shi from the Department of Authentication of Chinese Medicines in Beijing University of Chinese Medicine. All of them (ZZX 2016-001, 002, …, 010) were stored in the Herbarium of School of Chinese Materia Medica, Beijing University of Chinese Medicine.

Reference standards of chlorogenic acid, cryptochlorogenic acid, neochlorogenic acid, hesperidin, isochlorogenic acid A, isochlorogenic acid B isochlorogenic acid C were purchased from the Chinese Institute for the Control of Pharmaceutical and Biological Products (Beijing, China). The purities of all of the standards were greater than or equal to 98%. Norepinephrine (NE), dopamine (DA), 3,4-dihydroxyphenylacetic acid (DOPAC) and homovanillic acid (HVA), 5-hydroxytryptamine (5-HT), 5-hydroxy-3-indoleacetic acid (5-HIAA) were purchased from Sigma (St. Louis, MO, USA). The elisa kits of corticotropin releasing factor (CRF), adrenocorti cotrophic hormone (ACTH) and cortisol (CORT) were obtained from Bluegene Biotech Co., Ltd. (Shanghai, China). Diazepam was provided by Tianjin Lisheng Pharmaceutical Co, Ltd. (Tianjin, China) and HPLC grade methanol and acetonitrile were provided by Aladdin (Shanghai, China). All the other reagents were of analytical grade and were purchased from Beijing chemical factory (Beijing, China).

### 4.3. Sample Preparation

All the ten ZZX samples are naturally dried and finely pulverized, separately. Powder between the 24 and 65 mesh was weighed and extracted by 35% ethanol (250 g/2500 mL; reflux, 1 h × 3). The ZZX extractive were filtered by six layers of gauze and dried under reduced pressure at a temperature <80 °C.

### 4.4. HPLC Analysis

#### 4.4.1. Preparation of the Samples and Standard Solutions

The dried powder of ZZX extractive, equivalent to 0.3 g of crude drug, was dissolved in 10 mL MeOH to obtain the sample solution with a concentration of 0.03 g /mL. The standard solution of the mixture of seven reference compounds (chlorogenic acid, cryptochlorogenic acid, neochlorogenic acid, hesperidin, isochlorogenic acid A, isochlorogenic acid B, isochlorogenic acid C) was prepared by dissolving accurately weighed standards in MeOH. The standard solutions were stored in a refrigerator at 4 °C.

#### 4.4.2. Chromatographic Conditions

HPLC-PDA analysis was performed using a Waters Acquity system equipped with a binary solvent delivery pump and an auto sampler, connected to Waters Empower 2 software. The absolute content determination was performed on a ZORBAX SB-C18 Agilent column (4.6 × 250 mm, 5 μm) at a column temperature of 25 °C. The flow rate was 1 mL/min, and the detection wave length was 285 nm. Mobile phase was a mixture of ultrapure water with 0.1% formic acid (A) and ACN (B). The following gradient program was used: 0–8 min, 2–5% (B); 8–12 min, 5% (B); 12–25 min, 5–12.5% (B); 25–47 min, 12.5–18% (B); 47–68 min, 18–30% (B); 68–73 min, 30–57.5% (B) followed by a reequilibration step of 5 min. Under this chromatographic condition, chlorogenic acid, cryptochlorogenic acid, neochlorogenic acid, hesperidin, isochlorogenic acid A, isochlorogenic acid B andisochlorogenic acid C were able to achieve better separation.

Prior to injection, all the standard and ZZX solutions were filtered through a 0.24 μm Millipore membrane. The injection volume was 10 μL.

#### 4.4.3. Method Validation

The method validation was based on the recent guidelines (ICH Harmonised Tripartite Guideline, 1994, 2006) on specificity, linearity, precision, accuracy and robustness.

#### 4.4.4. Similarity Analysis

The chemical fingerprint of ZZX samples collected from 10 different regions was analysed by Similarity Evaluation System for Chromatographic Fingerprint of Traditional Chinese Medicine composed by Chinese Pharmacopoeia Committee (Version 2012 A) (Beijing, China). Using the median method, the similarity degrees of ZZX were calculated by this software. 

### 4.5. Anxiety Model

#### 4.5.1. Animals

A total of 104 Sprague-Dawley rats, aged 60 days and weighing 150–170 g, were purchased from Laboratory Animal Center of the Academy of Military Medical Sciences (Beijing, China, Certificate no.: SCXK-2016-0006). The first day of arrival, the rats were individually caged in a controlled environment (22 ± 2 °C; 50% RH) and were maintained on a 12:12 h light/dark cycle (light phase: 07:00–19:00). They were also allowed to eat and drink freely. Rats were habituated to the laboratory environment for 1 week and gently handled each day. Behavior experiments were performed between 09:00 and 15:00. The experimental procedures were approved by the Institutional Animal Care and Use Committee of the Institute of Psychology of the Chinese Academy of Sciences and in accordance with the National Institutes of Health Guide for Care and Use of Laboratory Animals.

#### 4.5.2. Procedure

**Figure 11:**
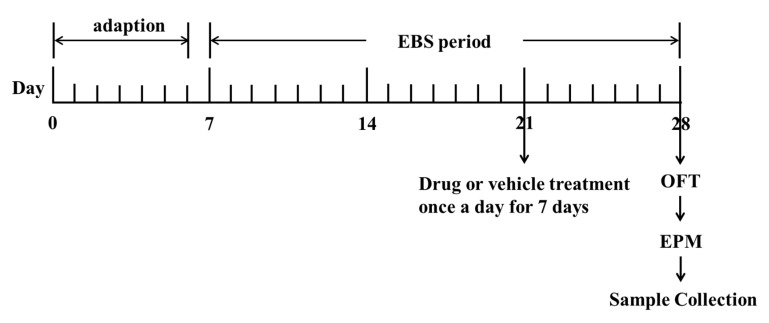
The flow chart of animal experiments.

The methods and dosages used in this study are all referred to in our previous studies and some adjustments were made [19]. Diazepam at the dose of 1 mg/kg was given in the DZP group. The dosage of ZZX in each group was 1.2 g/kg (Table 6). The control group and empty bottle stress model group (EBS group) were given physiological saline as placebo. All drugs mentioned were dissolved in physiological saline and prepared before use. The volume of administration was 1 mL/100 g of body weight. All rats were given drugs orally once a day for 7 days (Figure 11). Behavioral tests were conducted 1 h after the last administration. After the completion of behavioral tests, rats were decapitated immediately to obtain brain and blood samples.

#### 4.5.3. Empty Bottle Stimulation (EBS)

The EBS protocol was performed following the procedure described in the previous study, and simple modifications had been made [19,56] (Figure 11). A total of 21 days were taken to conduct the empty bottle stimulation anxiety model. For the first day after 1 week of acclimatization (day 7), except for the control group, rats in other groups were trained to drink water at 09:00 h to 09:10 h and 21:00 h to 21:10 h by allowing them access to water bottles only during these time periods for 1 week. After 1 week of training to drink water at fixation time (day 14), rats in the EBS group; DZP group; ZZX1-10 groups were randomly given empty bottles during one of the two watering periods for 2 weeks to induce anxious emotion. There was no restriction on the rats in the control group during all the time (Table 6). 

### 4.6. Behavioral Tests

#### 4.6.1. Open Field Test (OFT)

Rats were placed in the testing room, which is soundproofed, 1h before the test. The OFT apparatus was a 180 cm diameter cylinder with 60 cm high walls. The center of the bottom of the apparatus had a 52 cm diameter section, as previously described [57]. The rats were placed into the same point of the field and were allowed to freely explore for 5 min. During this 5 min, total distance traveled, time spent in the central area, and the number of central entries were recorded by an automated video-tracking system (SMART, Panlab, Barcelona, Spain). After each rat, the apparatus was quickly cleaned with 75% methanol.

#### 4.6.2. Elevated Plus Maze (EPM)

The elevated plus maze was made of black plexiglas and consisted of two open arms (50.8 cm × 10.2 cm × 1.3 cm) and two closed arms (50.8 cm × 10.2 cm × 40.6 cm) that extended from a central platform (10.2 cm × 10.2 cm). The maze was elevated to a height of 72.4 cm above the floor [57]. After the OFT, rats were gently transported to the EPM room. After 5 min of acclimatization, rats were placed on the central square, separately, facing one of the open arms. In the next 5 min, rats were allowed to explore the maze freely. It was counted as an arm entry when all four of the rat’s paws were entered into an arm. The time spent on both arms, and the number of entries into them were recorded by an automated video-tracking system (SMART, Panlab, Barcelona, Spain). After each rat, the apparatus was quickly cleaned with 75% methanol.

### 4.7. Detection of CRF, ACTH and CORT in Serum

Rats were anesthetized with 10% chloral hydrate after behavior tests. Blood samples were collected in vacuum blood collection tube through abdominal aortakept. Immediately, the blood samples were centrifuged at 3000 r⋅min^−1^ for 15 min at 4 °C. The supernatants were collected and stored at −80 °C until analysis. The contents of CRF, ACTH, and CORT were determined by enzyme-linked immunosorbent assay kits according to the manufacturer’s instructions. The absorbance of each sample was measured at a wavelength of 450 nm (ELx800, BioTek, Winooski, VT, USA). The results of CRF and ACTH were presented as pg/L, and the results of CORT were presented as ng/mL.

### 4.8. Detection of Monoamines and Metabolites

After blood collection, the rats’ brain were quickly removed and dissected on the ice plate. The cerebral cortex and hippocampus were weighed and homogenized respectively in 0.10 M perchloric acid by Polytron (Swedesboro, NJ, USA) homogenizer. Then the homogenates were centrifuged at 1200 r/min for 10 min at 4 °C. The supernatants were collected and stored at −80 °C until use. The concentration of monoamine and metabolites (NE, DA, DOPAC, HVA, 5-HT, 5-HIAA) were determined by HPLC coupled with an electrochemical detector (Model 2465, Waters, Milford, MA, USA) as described previously [58]. The HPLC system included a column (2.1 mm × 150 mm, 3 μm, Waters Atlantis). The mobile phase was 50 mM citric acid, 0.3 mM Na_2_-EDTA, 1.8 m Mdibutylamine, and 4% methanol (pH 3.5). The flow rate was 0.35 mL/min, and the detector potential was +0.75V, with an injection volume 20 μL. The levels of monoamines in the samples were expressed as nanograms per gram fresh weight of tissue.

### 4.9. Partial Least Squares Regression (PLSR) Model

PLSR is a multivariate calibration model mainly used to establish the regression equation of independent (X) and dependent (Y) variables [33]. If there was M principal components extracted from X, and there was N latent variables in each principal component. By establishing the multivariate linear regression equation of latent variables of X and Y, we can explain the inner relationship between X and Y [32]. In order to discover the bioactive chemical markers of ZZX, the relationship between chemical information (X: the contents of common compounds or common peak areas in chromatographic fingerprints) and pharmacodynamic information (Y: normalizedanti-anxiety efficacy index) was modeled by PLSR using SIMCA-P + 12.0 Demo. The principal analysis on each compound was performed automatically by the software. By manually adding the factor numbers, the explanatory abilities of the principal components to independent variables and dependent variables were both >90%. At the same time, the regression coefficients were recorded. The predominant chemical markers of ZZX affecting the pharmacodynamics index were determined by the sizes of the regression coefficients.

### 4.10. Statistical Analysis

All behavioral and physiological results were expressed as means ± standard error of the mean (SEM for short) individual value of rats from each group. Statistical analysis was performed using one-way analysis of variance (ANOVA), followed by the Student-Newman-Keuls post hoc test and Graph Pad Prism 5.0 software (Graphpad Sofware Inc, La Jolla, CA, USA). In cases of significant variation, the individual values were compared using Dunnett’s test. Values of *p* < 0.05 were considered statistically significant. 

## 5. Conclusions

In our study, the chemical fingerprint of ZZX samples collected from 10 different regions was determined by HPLC-PDA. Subsequently, the anti-anxiety effects of ZZX on the empty bottle stimulated rats were examined through the OFT and EPM. The fingerprint-efficacy study between the chemical fingerprint of ZZX and its anti-anxiety effect was accomplished by PLSR. We came to the conclusion that hesperidin, isochlorogenic acid A, isochlorogenic acid B andisochlorogenic acid C might be the bioactive chemical markers of ZZX. The fingerprint-efficacy method provided a feasible way and some elicitation for the quality control of ZZX and so as the other TCM. 

## Figures and Tables

**Table 1:** Repeatabilities, stabilities and recoveries of the 10 compounds in ZZX.

Analyte	Degree of Precision (*n* = 6)		Repeatability (*n* = 6)		Stability (0–12 h)		Recovery (*n* = 3)	
	Mean ± SD (Relative Peak Area)	RSD (%)	Mean ± SD (Relative Peak Area)	RSD (%)	Mean ± SD (Relative Peak Area)	RSD (%)	Mean (%)	RSD (%)
Neochlorogenic acid	0.1248 ± 0.0019	1.44	0.1206 ± 0.0030	2.51	0.1245 ± 0.0012	0.98	98.53	3.36
Unknown compound X	0.0462 ± 0.0010	2.01	0.0534 ± 0.0015	2.82	0.0452 ± 0.0006	1.25	-	-
Chlorogenic acid	0.0729 ± 0.0009	1.10	0.0766 ± 0.0008	1.08	0.0737 ± 0.0006	0.88	101.61	4.76
Cryptochlorogenin acid	0.0354 ± 0.0009	2.26	0.0383 ± 0.0006	1.71	0.0366 ± 0.0008	2.03	96.81	1.88
Unknown compound Y	1.0000 ± 0.0000	0.00	1.0000 ± 0.0000	0.00	1.0000 ± 0.0000	0.00	-	-
Unknown compound Z	0.1051 ± 0.0025	2.12	0.1028 ± 0.0004	0.42	0.1019 ± 0.0026	2.57	-	-
Isochlorogenic acid B	0.0493 ± 0.0009	1.62	0.0507 ± 0.0004	0.84	0.0480 ± 0.0010	2.04	101.52	3.28
Isochlorogenic acid A	0.0697 ± 0.0012	1.62	0.0815 ± 0.0005	0.54	0.0678 ± 0.0011	1.53	97.13	2.33
Hesperidin	0.1215 ± 0.0003	0.24	0.1290 ± 0.0013	1.04	0.1214 ± 0.0005	0.45	98.97	4.62
Isochlorogenic acid C	0.3938 ± 0.0044	1.02	0.4395 ± 0.0132	3.00	0.3939 ± 0.0027	0.68	95.77	1.01

SD, standard deviation; RSD, relative standard.

**Table 2:** Results of the analyses of linearity of calibration curves and sensitivities for the compounds in ZZX.

Analyte	tR (min)	Regression Equation	Regression Coefficient (r)	Linear Range (mg/mL)
Neochlorogenic acid	17.8	y = 28,912x + 57,452	0.9997	0.0242–0.1210
Chlorogenic acid	24.6	y = 26,703x − 772,245	0.9990	0.0627–0.8350
Cryptochlorogenin acid	25.9	y = 22,115x + 96,024	0.9991	0.0394–0.1972
Isochlorogenic acid B	48.7	y = 39,252x − 120,299	0.9994	0.0370–0.1852
Isochlorogenic acid A	50.9	y = 35,820x + 16,495	0.9990	0.0132–0.0660
Hesperidin	53.3	y = 34,376x + 4643.9	0.9998	0.0276–0.1381
Isochlorogenic acid C	55.1	y = 38,551x + 181,872	0.9991	0.0981–0.4905

**Table 3:** Contents or peak area of 10 common compounds in 10 batches of ZZX samples.

Analyte	ZZX-1	ZZX-2	ZZX-3	ZZX-4	ZZX-5	ZZX-6	ZZX-7	ZZX-8	ZZX-9	ZZX-10
Neochlorogenic acid (%)	0.0386	0.0506	0.0352	0.0368	0.0583	0.0353	0.0352	0.0425	0.0422	0.0396
Chlorogenicacid (%)	0.6071	1.5201	1.4152	1.5719	0.3970	1.3043	1.1137	1.0294	1.9673	1.9399
Cryptochlorogeninacid (%)	0.0362	0.0943	0.1087	0.1115	0.1376	0.0918	0.0725	0.0866	0.1468	0.1281
Isochlorogenic acid B (%)	0.0216	0.1905	0.0852	0.0495	0.0153	0.1475	0.0177	0.0646	0.0282	0.0574
Isochlorogenic acid A (%)	0.0176	0.5982	0.1623	0.0938	0.0070	0.4148	0.0175	0.1594	0.0252	0.1387
Hesperidin (%)	0.1840	0.2550	0.2159	0.2005	0.0639	0.2661	0.2389	0.3022	0.1604	0.1711
Isochlorogenic acid C (%)	0.0186	0.5713	0.2014	0.1049	0.0000	0.4217	0.0181	0.1563	0.0410	0.1239
Unknown compound X (Peak area)	172,864	514,637	579,261	788,200	1,313,283	658,845	1,740,288	921,530	1,350,526	813,070
Unknown compound Y (Peak area)	507,840	1,403,493	454,632	1,264,334	888,041	1,136,253	1,121,121	534,964	886,367	388,215
Unknown compound Z (Peak area)	425,946	1,418,969	591,482	1,022,988	848,343	1,042,095	1,110,699	765,774	912,019	503,629

**Table 4:** Effect of ZZX on the monoamines neurotransmitters and their metabolites in the hippocampus of rats.

Groups	NE (ng/g)	DOPAC (ng/g)	DA (ng/g)	5-HIAA (ng/g)	HVA (ng/g)	5-HT (ng/g)
**Control**	399.84 ± 63.79	247.60 ± 44.55	26.28 ± 8.30	184.16 ± 25.76	46.97 ± 11.24	56.06 ± 14.97
**EBS**	592.94 ± 58.82 ^#^	353.85 ± 45.16 ^##^	74.20 ± 22.61 ^##^	271.25 ± 56.02 ^##^	79.16 ± 21.88 ^##^	129.41 ± 39.42 ^#^
**DZP**	418.5 ± 79.19 *	262.67 ± 28.59 *	33.08 ± 11.51 **	185.71 ± 20.83 **	54.12 ± 3.83 *	59.30 ± 12.32 **
**ZZX-1**	514.18 ± 107.59	290.69 ± 76.92	41.74 ± 28.95	258.18 ± 76.81	63.12 ± 14.25	78.38 ± 47.19
**ZZX-2**	365.22 ± 113.11 *	257.44 ± 57.79 *	35.34 ± 18.11 *	241.57 ± 68.98 *	38.97 ± 11.61 *	81.48 ± 15.68 **
**ZZX-3**	388.25 ± 111.54 *	279.56 ± 94.88 *	29.03 ± 19.17 **	214.19 ± 42.40 *	51.46 ± 29.88 *	76.87 ± 19.53 **
**ZZX-4**	468.0 ± 128.74	311.05 ± 91.03	57.09 ± 42.65	214.29 ± 73.41 *	47.53 ± 17.03 *	65.42 ± 20.48 **
**ZZX-5**	479.23 ± 101.04	304.94 ± 79.96	58.17 ± 25.19	279.48 ± 52.64	64.06 ± 15.87	85.13 ± 38.76
**ZZX-6**	470.13 ± 121.40 *	248.43 ± 21.50**	34.85 ± 19.95 **	168.38 ± 37.69 **	60.65 ± 11.24 **	70.41 ± 35.15 *
**ZZX-7**	546.36 ± 85.83	299.22 ± 53.40	44.33 ± 26.78 *	181.71 ± 22.54 *	57.89 ± 13.29	76.47 ± 16.45
**ZZX-8**	442.69 ± 138.78 **	271.39 ± 51.85 *	38.24 ± 16.40 *	163.16 ± 35.11 **	58.20 ± 10.95 *	62.55 ± 36.19 *
**ZZX-9**	598.26 ± 138.24	237.77 ± 40.21 *	35.31 ± 17.82 **	150.58 ± 42.60 *	59.88 ± 10.95 **	77.11 ± 34.85 *
**ZZX-10**	532.55 ± 121.78 **	309.96 ± 49.89	61.64 ± 25.37	170.47 ± 31.43 *	50.63 ± 11.24 **	60.90 ± 16.48 **

Values are expressed as the mean ± S.E.M. ^#^ represent *p* < 0.05 compared to the control group; ^##^ represent *p* < 0.01 compared to the control group; * represent *p* < 0.05 compared to the EBS/ZZX group; ** represent *p* < 0.01 compared to the EBS/ZZX group. One-way ANOVA with Student-Newman-Keuls post hoc test.

**Table 5:** Effect of ZZX on the monoamines neurotransmitters and their metabolites in the whole cerebral cortex of rats.

Groups	NE (ng/g)	DOPAC (ng/g)	DA (ng/g)	5-HIAA (ng/g)	HVA (ng/g)	5-HT (ng/g)
**Control**	371.44 ± 49.10	335.56 ± 56.02	708.48 ± 198.70	102.01 ± 20.21	131.98 ± 45.23	81.63 ± 23.93
**EBS**	527.92 ± 93.21 ^#^	477.01 ± 81.89	1148.85 ± 140.90 ^##^	168.61 ± 33.50 ^##^	201.09 ± 11.64	146.45 ± 31.74 ^##^
**DZP**	403.83 ± 66.98 *	379.02 ± 39.69	778.52 ± 165.11 *	113.40 ± 20.55 **	138.23 ± 15.01	99.82 ± 14.83 **
**ZZX-1**	339.35 ± 92.28 **	389.05 ± 97.95	1015.91 ± 352.17	121.34 ± 70.99	155.91 ± 49.49	124.45 ± 26.81
**ZZX-2**	349.29 ± 185.38 *	366.22 ± 105.52	685.24 ± 424.67 *	100.85 ± 18.90 *	143.41 ± 70.28	94.10 ± 36.21 **
**ZZX-3**	442.65 ± 100.13 *	373.37 ± 104.25	905.71 ± 227.50 *	122.04 ± 42.90 *	147.66 ± 48	130.71 ± 25.89
**ZZX-4**	456.23 ± 116.28 *	361.80 ± 113.46	909.67 ± 200.29 *	102.75+42.15 *	165.92 ± 74.54	107.45 ± 15.33 **
**ZZX-5**	459.17 ± 87.08	407.63 ± 77.75	975.49 ± 243.88	103.56 ± 69.39	148.68 ± 68.60	109.48 ± 21.35 **
**ZZX-6**	350.44 ± 116.39 *	378.41 ± 52.44	770.81 ± 313.60 *	121.97 ± 33.94 *	151.31 ± 45.49	108.58 ± 33.10 *
**ZZX-7**	381.71 ± 113.65	393.95 ± 66.10	809.32 ± 262.26 *	123.12 ± 32.43 *	147.85 ± 43.61	127.43 ± 38.67
**ZZX-8**	375.11 ± 65.88 *	367.00 ± 74.51	643.80 ± 319.77 *	123.58 ± 35.24 *	159.78 ± 54.19	98.21 ± 39.00 *
**ZZX-9**	339.75 ± 136.14	426.61 ± 62.73	810.72 ± 370.29	135.78 ± 44.43	140.49 ± 51.52	95.07 ± 38.75 *
**ZZX-10**	367.83 ± 146.72	402.34 ± 68.85	779.76 ± 221.48 *	142.82 ± 21.24	149.96 ± 33.45	107.74 ± 25.45 *

Values are expressed as the mean ± S.E.M. ^#^ represent *p* < 0.05 compared to the control group; ^##^ represent *p* < 0.01 compared to the control group; * represent *p* < 0.05 compared to the EBS/ZZX group; ** represent *p* < 0.01 compared to the EBS/ZZX group. One-way ANOVA with Student-Newman-Keuls post hoc test.

**Table 6:** Experimental groups and procedure.

Group	Medicine and Sources	Model	Dose	Animal
Control	N.S.	-	-	8
EBS	N.S.	Empty Bottle Stimulation (EBS)	-	8
DZP	DZP		1 mg/kg	8
ZZX-1	ZZX from Dali Yunnan		1.2 g/kg	8
ZZX-2	ZZX from Anguo Hebei		1.2 g/kg	8
ZZX-3	ZZX from Baoshan Yunnan		1.2 g/kg	8
ZZX-4	ZZX from Lotus Pond in Sichuan		1.2 g/kg	8
ZZX-5	ZZX from Yongping Yunnan		1.2 g/kg	8
ZZX-6	ZZX from Guizhou		1.2 g/kg	8
ZZX-7	ZZX from Shanxi		1.2 g/kg	8
ZZX-8	ZZX from Jiuding Mount in Sichuan		1.2 g/kg	8
ZZX-9	ZZX from Chongqing		1.2 g/kg	8
ZZX-10	ZZX from Guangxi		1.2 g/kg	8

N.S: normal saline; DZP: diazepam.

## Abbreviations

ZZX	Zhi zhu xiang
TCM	Traditional Chinese Medicine
HPLC	High Performance Liquid Chromatography
OFT	Open Field Test
EPM	Elevated Plus Maze Test
CRF	Corticotropin releasing factor
ACTH	Adrenocorti cotrophic hormone
CORT	Cortisol
ELISA	Enzyme-linked immune sorbent assay
NE	Norepinephrine
DA	Dopamine
DOPAC	3,4-dihydroxyphenylacetic acid
HVA	Homovanillic acid
HT	5-hydroxytryptamine
5-HIAA	5-hydroxy-3-indoleacetic acid
PLSR	Partial least squares regression
HPA	The hypothalamic-pituitary-adrenal axis
EBS	Empty bottle stimulation
